# Graphene-Based Nanomaterials for Tissue Engineering in the Dental Field

**DOI:** 10.3390/nano8050349

**Published:** 2018-05-20

**Authors:** Riccardo Guazzo, Chiara Gardin, Gloria Bellin, Luca Sbricoli, Letizia Ferroni, Francesco Saverio Ludovichetti, Adriano Piattelli, Iulian Antoniac, Eriberto Bressan, Barbara Zavan

**Affiliations:** 1 Department of Neurosciences, Institute of Clinical Dentistry, University of Padova, 35128 Padova, Italy; riccardo.guazzo@unipd.it (R.G.); luca.sbricoli@unipd.it (L.S.); francesco.ludovichetti@unipd.it (F.S.L.); eriberto.bressan@unipd.it (E.B.); 2 Department of Biomedical Sciences, University of Padova, 35131 Padova, Italy; chiara.gardin@unipd.it (C.G.); gloria.bellin@gmail.com (G.B.); 3 Maria Pia Hospital, GVM Care & Research, 10132 Torino, Italy; 4 Department of Medical, Oral and Biotechnological Sciences, University of Chieti-Pescara, 66100 Chieti, Italy; apiattelli@unich.it; 5 Department Materials Science and Engineering, University Politehnica of Bucharest, 060032 Bucharest, Romania; antoniac.iulian@gmail.com; 6 Maria Cecilia Hospital, GVM Care & Research, 48033 Ravenna, Italy

**Keywords:** graphene, nanomaterials, dental stem cells, antibacterial activity, dental implant, bone regeneration

## Abstract

The world of dentistry is approaching graphene-based nanomaterials as substitutes for tissue engineering. Apart from its exceptional mechanical strength, electrical conductivity and thermal stability, graphene and its derivatives can be functionalized with several bioactive molecules. They can also be incorporated into different scaffolds used in regenerative dentistry, generating nanocomposites with improved characteristics. This review presents the state of the art of graphene-based nanomaterial applications in the dental field. We first discuss the interactions between cells and graphene, summarizing the available in vitro and in vivo studies concerning graphene biocompatibility and cytotoxicity. We then highlight the role of graphene-based nanomaterials in stem cell control, in terms of adhesion, proliferation and differentiation. Particular attention will be given to stem cells of dental origin, such as those isolated from dental pulp, periodontal ligament or dental follicle. The review then discusses the interactions between graphene-based nanomaterials with cells of the immune system; we also focus on the antibacterial activity of graphene nanomaterials. In the last section, we offer our perspectives on the various opportunities facing the use of graphene and its derivatives in associations with titanium dental implants, membranes for bone regeneration, resins, cements and adhesives as well as for tooth-whitening procedures.

## 1. Introduction

Tissue engineering is an interdisciplinary science which aims at developing biological substitutes to restore, maintain, or improve tissue function by using a combination of cells, scaffolds and suitable biochemical factors [1]. Scaffolds in particular represent the key element in tissue engineering research, whose role is not only to provide the appropriate environment for specific cells but also to retain growth and nutrition factors for cellular migration, adhesion, growth and differentiation [2].

Over the years, several natural and synthetic three-dimensional (3D) scaffolds have been successfully developed and employed for various tissues, such as skin [3], cartilage [4], muscle [5], vasculature [6] and bone [7]. With regard to dental tissues, regeneration is very challenging and requires thorough understanding of biological events at the cellular and molecular level [8]. Tooth indeed is one of the most difficult tissues to treat due to its heterogeneous and dynamic anatomical structure which includes the vital dentin-pulp complex, cementum, periodontal ligament, alveolar bone and enamel [9]. Furthermore, dental tissues show a limited and variable degree for self-repair as a result of injury or disease: cementum, for example, has a very slow regenerative capacity, whereas enamel regeneration is not possible. Dentin can regenerate, while dental pulp has a partial regeneration capacity as it is enclosed in dentin and has limited apical blood supply. In contrast, alveolar bone exhibits rapid turnover in response to mechanical stimulation [10].

In the last decade, many advances have been made in the dental tissue engineering field aimed at the regeneration of dental pulp [11], periodontal ligament [12,13], dentin [14], enamel [15] and integrated tooth tissues [16,17], which have seen the interplay of stem cells, growth factors and scaffolds. Scaffolds including collagen [18], polymers [19], self-assembling peptides [20], or silk [21] have been used for dental tissues regeneration. A novel strategy that may improve the success of scaffold therapy is represented by nanosized materials [22]. Nanomaterials possess exciting physicochemical and biological properties for biomedical applications due to their small size, large surface area and ability to interact with cells, promoting their adhesion, migration, proliferation and differentiation [23].

**Figure 1:**
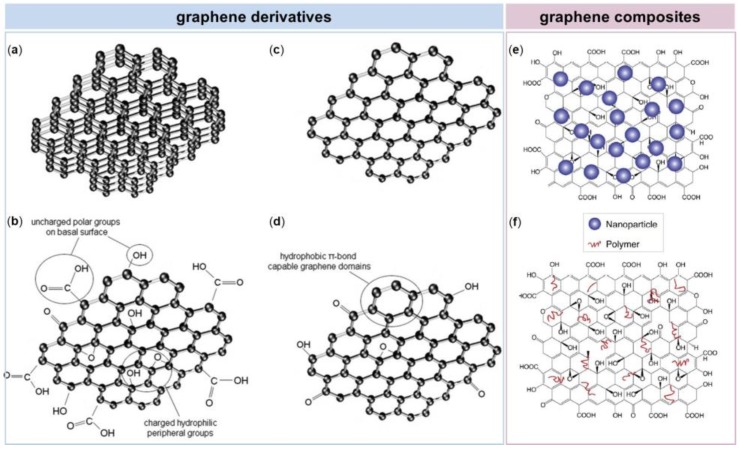
Schematic representation of different graphene-based nanomaterials. (**a**) Few-Layered Graphene (FLG), (**b**) Graphene Oxide (GO), (**c**) graphene nanosheets and (**d**) reduced Graphene Oxide (rGO) belong to the graphene derivatives group; (**e**) GO nanoparticle composite and (**f**) GO polymer composite are composites of graphene. Reproduced with permissions from [25,26].

In the last few years, graphene and its derivatives have emerged as a new class of nanomaterials [24]. Graphene is a carbon-based flat monolayer, arranged in a two-dimensional hexagonal structure, with unique mechanical, electrochemical and physical properties. The graphene family nanomaterials include several graphene derivatives, such as Few-Layered Graphene (FLG), ultrathin graphite, Graphene Oxide (GO), reduced Graphene Oxide (rGO) and graphene nanosheets [25]. These differ from each other in terms of surface properties, number of layers and size. In addition to the above graphene derivatives, graphene family also comprises graphene-based composites, which derive from functionalization of graphene with polymers, small molecules, or nanoparticles through covalent or noncovalent interactions [26] (Figure 1). Graphene surface functionalization with molecules of diverse nature allows the development of different devices, that can enhance or alter the properties required for specific application.

Since its discovery in 2004 [27], many applications have been explored for graphene, ranging from electronic and optoelectronic devices to photoconductive materials. However, only in 2008 graphene was introduced for the first time in the field of biomedical sciences [28] and widely used in biomedical applications such as bioelectronics, bioimaging, drug delivery and tissue engineering [24].

The present review intends to provide the reader an overview of the current state of the art of the graphene-based nanomaterials in tissue engineering in dentistry. Different aspects of the graphene-based nanomaterials will be discussed in detail. First, we highlight the role of stem cells and their interactions with graphene nanomaterials. We consider the influence of graphene-based nanomaterials both on biocompatibility, cytotoxicity and differentiation properties of stem cells. In this context, particular attention will be given to stem cells of dental origin. The interactions with immune cells and antibacterial properties of graphene, which are critical aspects to consider in the hostile environment of the oral cavity, are subsequently discussed. Finally, applications of graphene-based nanomaterials on dental implants, membranes, resins, cements and adhesives as well as on teeth-whitening are extensively presented.

## 2. Graphene-Based Nanomaterials and Interactions with Cells

The first aspect to consider when developing a new nanomaterial for biomedical applications is its biocompatibility. Excellent biocompatibility is indeed essential in order to avoid any adverse effect of a material in living tissues [29]. Apart from being biocompatible, scaffolds should direct the successful transformation of stem cells into cells and tissues with morphological features and physiological functions similar to those in vivo. In other words, tissue engineering techniques can be considered efficient when they obtain stem cell differentiation into the desired tissue lineage [30]. Furthermore, an ideal scaffold should not evoke an inflammatory response and desirably inhibit bacterial growth on the surfaces. This feature is very critical when considering the environment of the oral cavity, which is known to be an important site for bacterial biofilm formation. In the following paragraphs, the effects of incorporation of graphene-based nanomaterials into different scaffolds, in relation to the issues described above, will be discussed in detail.

### 2.1. Graphene Biocompatibility In Vitro and In Vivo

Graphene-based nanomaterials are rapidly spreading as scaffolds in tissue engineering applications, also in the dental field; consequently, assessments of their biocompatibility and cytotoxicity have to be done. Dosages, viability assays, type of nanomaterials, type of cells and uses need to be evaluated before developing a new reliable graphene-based nanomaterial.

Over the years, biocompatibility of graphene and its derivatives has been largely discussed among authors. The comprehension of the toxicological potential of graphene-based nanomaterials, indeed, has to be carefully evaluated in order not to interrupt future in vivo studies and translational efforts [31]. Accumulating evidence suggests that the cytotoxicity of graphene and its derivatives is influenced by numerous factors, such as their concentration, shape, size, dispersibility, surface functionalization and that the common mechanisms describing the cytotoxicity of graphene-based nanomaterials include Reactive Oxygen Species (ROS) production and cell membrane damage [32,33].

The 3-(4,5-Dimethylthiazol-2-yl)-2, 5-Diphenyltetrazolium bromide (MTT) assay, which measures the mitochondria function of the cells, is one of the preferred method for evaluating the effect of nanomaterials in cell culture [34]. In one of the pioneering works, the in vitro toxicity of FLG sheets was compared to that of Single Wall Carbon Nanotubes (SWCNT) by using neuronal PC12 cells [32]. The authors found that the toxicity of these two nanomaterials of relatively identical chemical structure was concentration-dependent, but, interestingly, these showed a different pattern of toxicity. FLG sheets resulted more toxic than SWCNT at low concentrations; whereas at higher concentrations graphene showed a lower activity, thus reversing its cytotoxic effect. These results can be primarily explained by the different shape of graphene sheets and SWCNT. Indeed, apart from concentration, the shape of nanomaterials plays an extremely important role, determining how these interact with cells and with potentially other biological systems.

Shortly after, another work evaluated the toxicity and biocompatibility of GO to human lung A549 cells, a widely used cell line for toxicity studies [33]. Compared to the previous study, the authors found that GO has much lower toxicity, as indicated by the results of the viability assay and Lactate Dehydrogenase (LDH) leakage assay. Together with MTT, the LDH assay, which measures enzyme release resulting from membrane damage, is another reliable and widely accepted test for evaluating materials toxicity [35]. The results of this work can be explained assuming that the cytotoxicity of nanomaterials is also heavily influenced by their surface functionalization. In this case, GO functionalization is higher than that of FLG sheets due to the presence of oxygenated functional groups. GO is indeed the oxidized derivative of graphene, produced by the oxidation of bulk graphite powders via chemical oxidation processes. It contains several reactive oxygen functional groups, such as epoxide, carboxyl and hydroxyl groups, which stabilizes the GO sheets on water, thus making them more hydrophilic [36].

Another work that stresses this concept is the one by Sasidharan and colleagues [37]. They found that, while pristine graphene accumulated on the cell membrane causing high oxidative stress leading to apoptosis, hydrophilic functionalized graphene, although internalized by the cells, did not cause any toxicity. The potential cytotoxicity of graphene-based nanomaterials is, therefore, highly dependent on their functionalization degree, as also established in the study of Das and coworkers [38]. When comparing the toxicity of GO and rGO, the first was found to be more toxic than rGO of same size on all the three cell lines used in the study. rGO is the reduced form of graphene; it is mainly produced to restore the electrical conductivity and optical absorbance of GO while reducing the oxygen content, surface charge and hydrophilicity [39]. Authors supposed that cytotoxicity was mediated by inducing ROS generation in the cells and that the presence of more reactive functional groups of GO would have a greater potential to interact with biological macromolecules compared with rGO. Nevertheless, by decreasing extent of oxygen functional group density on the GO surface, it was possible to reduce oxidative stress and consequently the nanomaterials toxicity.

**Figure 2:**
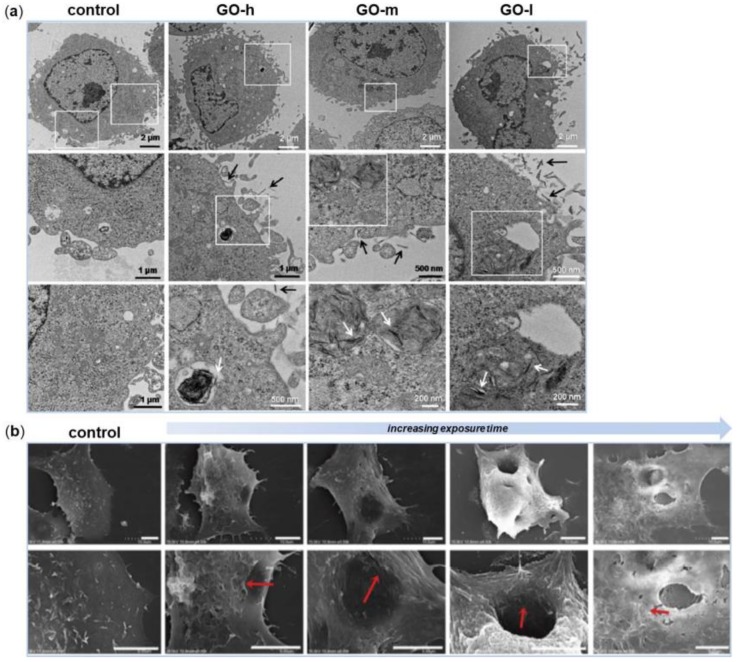
Morphological observations of cells after interactions with graphene-based nanomaterials. (**a**) Transmission Electron Microscopy (TEM) images of Mouse Embryo Fibroblasts (MEFs) treated with GO-high (GO-h), GO-medium (GO-m) and GO-low (GO-l) at 50 µg/mL for 24 h. On bottom, high-magnification images of the boxed-in photos on top are represented. The white and black arrows indicate GO aggregates inside and outside cells, respectively. (**b**) Scanning Electron Microscopy (SEM) images of cell membrane damage incurred by A549 cells as a result of GO nanosheets exposure observed during different phases of incubation. On bottom, high-magnification images of the boxed-in photos on top are represented. Reproduced with permissions from [40,42].

The physicochemical mechanism of the oxidation-state dependent cytotoxicity of GO was proposed recently by the group of Zhang [40]. They synthesized three GO samples with similar size distribution and solubility in water, but with different oxidation state; these samples were then tested on Mouse Embryo Fibroblasts (MEFs). First of all, their results suggested that 50 µg/mL may be a threshold for GO to exhibit toxicity on normal mammalian cells. Then, they found that the GO with lower degree of oxidation displayed stronger toxicity on MEFs and stimulated higher intracellular ROS production (Figure 2a). As resulted from Electron Spin Resonance (ESR) spectrometry analysis, the decrease in the oxidation state was correlated to a higher ability of the GO samples to promote H_2_O_2_ decomposition and ⋅OH formation. Among various types of ROS, ⋅OH is an extremely reactive free radical and indiscriminately induces oxidative damages on various biomolecules, including DNA, lipids and proteins [41].

Soon after, another distinct process contributing to the molecular basis of graphene cytotoxicity was unraveled by Duan and colleagues [42]. They demonstrated that GO nanosheets were responsible to induce pore formation on cell membranes. Molecular dynamics simulations revealed that the molecular mechanism for perforation was dependent on cooperative lipid extraction driven by several graphene nanosheets (Figure 2b).

With regard to in vivo experiments, there are few studies concerning the biocompatibility of graphene-based nanomaterials since most of the investigations have only focused on the in vitro aspect of research. Nevertheless, the in vitro cell culture environment is definitely different from the in vivo complex 3D physiological condition. In addition, typical cytotoxicity assays measure the effect only during the first 12–24 h after exposure to a toxic substance although many biological reactions in vivo continue beyond 24 h.

As for in vitro experiments, the in vivo effects of graphene-based nanomaterials were observed to be dependent on their concentration, physicochemical properties, exposure time, but also on administration route and on the characteristics of the animals used in the assays [43].

Wang and colleagues observed that intravenous injection of GO at 0.1 mg and 0.25 mg showed no obvious toxicity to mice, whereas almost half animal population died after administration of 0.4 mg GO [44]. All deaths occurred within days after injection of the GO. When investigating the effects of GO on organs of mice, the authors determined that graphene-based nanomaterials mainly accumulate in liver, spleen and lung after intravenous injection.

In another study, GO or pluronic-dispersed graphene was administered by intratracheal instillation to mice [45]. The authors found that GO induced acute lung injury that persisted for more than 21 days after administration. Lung inflammation was attributed to GO ability to increase the rate of mitochondrial respiration and the generation of ROS in cells, activating inflammatory and apoptotic pathways. On the contrary, toxicity was significantly reduced when the unoxidized graphene was well-dispersed with the block copolymer pluronic. These results demonstrate that graphene oxidation contributes to its pulmonary toxicity, whereas dispersion of pristine graphene in pluronic represents a safe way for handling and potential biomedical application of graphene.

Later, the group of Yang tested several Polyethylene Glycol (PEG) functionalized GO materials with varied sizes and surface chemistry after oral and intraperitoneal injection to mice [46]. The authors observed that few h after being administered by oral route, PEGylated GO derivatives were present in high quantities in stomach and intestine, but not in other major organs. However, only low levels were detected in the organs after 1 day and no trace was detected after 1 week. In contrast, high accumulation of PEGylated GO derivatives was observed in the liver and spleen after intraperitoneal injection, but their levels gradually decreased.

Considering certain inconsistencies and contradictory results in several studies, generalizations about graphene toxicity should be avoided. Indeed, it is generally accepted that the in vitro and in vivo behaviors of graphene-based nanomaterials are closely associated with their physicochemical properties, such as surface functionalization, coating, size and importantly, the administration routes. However, based on the studies presented in this section, we may summarize that the hydrophobic forms of graphene-based nanomaterials, which accumulate on the surface of cell membranes, are more toxic compared to the most hydrophilic ones. These, in fact, are more prone to infiltrate the cell membrane and to be internalized, as well as being removed from the application site. At the same time, when comparing GO with its reduced counterpart, rGO, the latter results less toxic than the hydrophilic form. Anyway, by controlling the GO reduction and maintaining the solubility, it is possible to minimize the GO toxicity and unravel the wide range of biomedical applications.

### 2.2. Graphene, Stem Cells and Osteogenesis

As mentioned before, one of the key characteristics of the ideal scaffold is an effective and controlled guidance of the proliferation and differentiation of stem cells into specific tissue lineages, either in the absence or presence of chemical inducers and growth factors. In the past few years, graphene-based nanomaterials have been increasingly used as scaffold materials for stem cell growth and differentiation. In the specific field of dental tissue engineering, most of the studies focused on evaluating the role of graphene in driving the osteogenic differentiation of stem cells. Indeed, bone regeneration plays a fundamental role in regenerative dentistry and how incorporation of graphene-based nanomaterials can enhance osteoconductivity through stimulating both cellular osteogenic differentiation and biomineralization have been extensively investigated [24].

In one of the first in vitro studies using stem cells, graphene produced by the Chemical Vapor Deposition (CVD) method and combined with different substrates was found to increase human Mesenchymal Stem Cells (MSCs) proliferation with no signs of cytotoxicity [47]. MSCs are one of the major progenitor cells achievable from a number of sources including adipose tissue, bone marrow and dental tissues [48]. Remarkably, graphene-coated scaffolds not only supported the growth and proliferation of human MSCs, but also accelerated their specific differentiation into osteoblasts, based on the level of the osteogenic marker osteocalcin and calcium mineralization level. The observed phenomena were obtained in the absence of additional growth factors and the acceleration rate was similar to that achieved with the use of typical osteogenic growth factors.

Starting from these results, another group investigated whether the graphene-induced osteogenesis of human MSCs was correlated to the chemical properties of graphene [49]. In the study, the effects of graphene and GO substrates were explored on both the osteogenic and adipogenic differentiation of human Bone Marrow MSCs (BM-MSCs). The results of the work revealed the ability of graphene to induce BM-MSCs differentiation into osteoblasts, probably by acting as a pre-concentration platform for dexamethasone and beta-glicerophosphate, typical osteogenic differentiation factors. In contrast, adipogenic differentiation resulted suppressed on graphene but strongly enhanced on GO substrates. In this case, the authors hypothesized that GO possesses high adsorption capacity of insulin, the key mediator for fatty acid synthesis.

**Figure 3:**
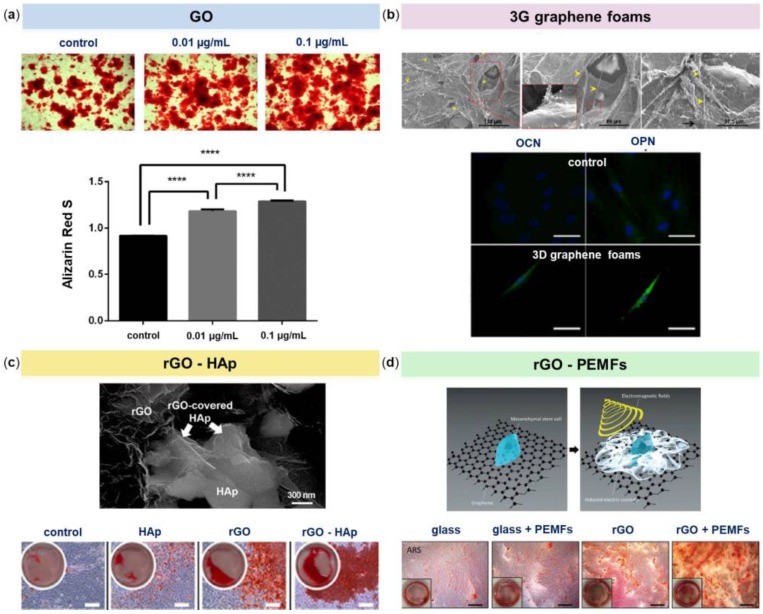
Effect of graphene-based nanomaterials on the osteogenic differentiation of Mesenchymal Stem Cells (MSCs). (**a**) Evaluation of matrix mineralization by means of Alizarin Red S (ARS) staining (top panel) and quantification (bottom panel). MSCs were grown for 21 days in osteogenic differentiation medium on GO nanosheets. The level of mineralization in the 0.1 μg/mL GO group was significantly higher than the other two conditions. (**b**) Human BM-MSCs cultured on 3D graphene foams for 4 days show protrusions up to 100 μm in length (yellow arrowheads) that extended from the cell bodies (black arrows), as evidenced by SEM images (top panel). These 3D substrates were also found to promote the expression of the osteogenic markers Osteocalcin (OCN) and Osteopontin (OPN), as displayed by immunofluorescence images (bottom panel). (**c**) SEM images of the rGO-coated Hap nanocomposites showing that Hydroxyapatite (HAp) particles were partly covered and interconnected by an network of rGO nanosheets (top panel). ARS staining performed at 21 days reveals that rGO-coated HAp nanocomposites significantly increase calcium deposits in MC3T3-E1 cells compared to the non-treated control and rGO or HAp alone (bottom panel). (**d**) Schematic representation of the study: human BM-MSCs were seeded onto rGO substrates, then exposed to Pulsed Electromagnetic Fields (PEMFs) (top panel). The rGO+PEMFs group exhibited the strongest staining as evidenced by ARS staining performed after 2 weeks from cells seeding. Reproduced with permissions from [50,51,53,62].

Other studies have further tested the effects of GO on MSCs growth and osteogenic differentiation and the results varied. In the interesting work of Wei and colleagues, the authors compared the cellular behaviors of BM-MSCs towards pristine GO nanosheets using two in vitro biomimetic culture methods, that imitate similar situations in vivo [50]. The sequential-seeding method would mimic the interaction between GO and established cells, whereas the co-seeding method resembles the interaction between GO and migrating cells. The authors found that both cell proliferation and differentiation were dependent on pristine GO nanosheets concentration and in vitro culture methods. In particular, BM-MSCs cultured with the sequential-seeding method resulted less vulnerable than those cultured with the co-seeding method, in which the cells had not yet adhered to the substrate. This seeding approach was beneficial for cells adhesion and proliferation when treated with 0.1 μg/mL of GO. Analogously, differentiation into osteoblasts was efficiently promoted when BM-MSCs were treated with this GO concentration, but only when osteogenic inducers were added to culture media (Figure 3a).

In recent years, also 3D graphene-based scaffolds have been actively explored and developed for the growth and differentiation of stem cells. The group of Crowder was the first to employ 3D graphene foams as substrates for human BM-MSCs culture [51]. When seeded onto these foams for 7 days, BM-MSCs were shown to maintain viability; in addition, the cells strongly expressed the osteogenic markers Osteocalcin (OCN) and Osteopontin (OPN), indicating their spontaneous osteogenic differentiation without the need for extrinsic biochemical factors (Figure 3b). It could be assumed that 3D graphene foams provided in vivo-like microenvironments which were conducive for the differentiation of stem cells.

Besides being used in their pristine form, graphene nanomaterials have also been combined with different polymers, nanofibers, or nanoparticles to create novel composites for controlling the growth and differentiation of stem cells. Hydroxyapatite (HAp), for example, is a calcium phosphate ceramic commonly used for bone repair or regeneration due to its chemical similarity to that of natural apatite in bones [52]. The recent work of Lee and colleagues demonstrated that rGO-coated HAp nanocomposites directed spontaneous differentiation of the murine preosteoblastic MC3T3-E1 cells towards bone lineage even in the absence of osteogenic differentiation factors [53]. Nevertheless, the osteogenic activity mediated by the rGO-coated HAp composites was further enhanced when these agents were added to the culture medium (Figure 3c). The authors concluded that, as the rGO-coated HAp composites showed to be potent inducers of spontaneous osteogenic differentiation of human MSCs, their application in the orthopedic and dental field may be determinant.

Natural bone is a composite material in which inorganic HAp nanocrystals are orderly embedded in an organic matrix made of collagen [54]. Collagen sponges are clinically approved scaffolds for bone regeneration [55]. These provide soft microenvironment to MSCs; however, stem cells preferentially differentiate to osteogenic lineage when cultured on mechanically stiff substrates. In order to overcome this problem, the group of Kang modified collagen sponge scaffolds by covalently incorporating GO flakes [56]. The covalent conjugation of GO flakes to collagen scaffolds increased the scaffold stiffness, which was comparable to that of pre-mineralized bone matrix and did not cause cytotoxicity of human MSCs. Importantly, when compared to non-modified collagen scaffolds, the GO-collagen sponges significantly enhanced the osteogenic differentiation of the cells. The authors hypothesized that the enhanced osteogenic differentiation was likely mediated by MSCs mechanosensing, since molecules that are involved in cell adhesion to stiff substrates, such as focal adhesions and cytoskeletal proteins, were either up-regulated or activated. In a following work of Nishida and coworkers [22], the effect of GO incorporation into 3D collagen sponges was examined in vitro and in vivo and compared to that observed with untreated collagen scaffolds. A previous study of the same group revealed clear morphological changes of collagen scaffolds after GO coating, that were dependent on GO concentration [57]. Notably, GO coating did not alter the porous structure of collagen sponges but reinforced their stability. Also, in their following work, the authors found that GO application improved the physical properties of collagen scaffold, such as compressive strength, enzyme resistance and adsorption of calcium and proteins. In addition, when GO scaffolds were implanted into a dog tooth extraction socket, the rate of new bone formation was higher than in the control group consisting of untreated collagen scaffolds, where most of the socket area was filled by connective tissue. These results were obtained by means of histomorphometric measurements and radiographic images taken 2 weeks after implantation.

Among others available materials, peptides containing the repeating arginine-glycine-aspartic acid (RGD) sequence, which is often found in Extracellular Matrix (ECM) proteins, have been widely applied to enhance cell adhesion to synthetic polymers or metallic surfaces [58,59]. In one of the latest studies, a 3D graphene/RGD peptide nanoisland composite was developed and tested on the osteogenesis of human Adipose-Derived Stem Cells (ADSCs) [60]. Human ADSCs represent excellent candidates being accessible for autologous transplantation and able to differentiate into osteogenic lineage for bone regeneration [61]. Also, in this work beta-glicerophosphate, dexamethasone and ascorbic acid were added to the culture medium for induction of osteogenesis. Such scaffolds were found to accelerate ADSCs osteoblastic differentiation, as revealed by gene expression results, Alkaline Phosphatase (ALP) activity measurements and evaluation of calcium deposition over a period of 4 weeks. Thus, the combination of RGD-containing peptides and GO substrates enhanced the adhesion strength of stem cells, ultimately resulting in increased osteogenic differentiation, as cell adhesion is the first step driving osteogenic lineage commitment. Taken together, the results of these studies would suggest that nanomodifications of different scaffolds using GO might provide good structure for colonization by several cell types.

One last work worth mentioning is the one of Lim and colleagues [62]. The authors used Pulsed Electromagnetic Fields (PEMFs) in combination with rGO substrates to evaluate the osteogenic differentiation of human alveolar BM-MSCs. PEMFs therapy is based on the physical effect sorted from the combination of an electric with a magnetic field, generating waves that propagate at the speed of light; and it has already been reported to strongly enhance the commitment of ADSCs into mature osteoblasts in vitro [63]. Cells cultured using osteogenic medium were exposed to 30 minutes daily treatments of 0.6 ± 0.05 mT and 50 Hz PEMFs for up to 2 weeks. After 7 days, rGO+PEMFs promoted the expression of ALP, an early marker of osteogenesis, whereas at week 2, the same scaffolds stimulated OPN, OCN and Runt-related transcription factor 2 (RUNX2) expressions as well as calcium deposition. In addition, DNA microarray analysis established that the combination of rGO and PEMFs increased the expression of genes related to ECM formation, membrane proteins and metabolism. The results of this work seem to demonstrate the synergic effects of rGO scaffolds and PEMFs on the osteogenesis of human alveolar BM-MSCs (Figure 3d).

All the works presented in this section demonstrate that, over the last few years, graphene has been found as one of the most promising biocompatible scaffolds for human MSCs adhesion, proliferation and differentiation, particularly towards the osteogenic lineage. These recent investigations clearly indicate a potential or active ability of graphene and its derivatives to induce the osteogenic differentiation of MSCs. Graphene alone seems to drive the spontaneous osteogenesis of stem cells; however, the presence of osteogenic inducers or growth factors in culture medium contributes in promoting this effect. Further summarizing the studies presented here, we may conclude that the association of graphene with stiff substrates, such as HAp, or with natural bone components, such as collagen, enhances the intrinsic properties of graphene in guiding the osteogenic differentiation of stem cells. Also mimicking the natural bone ECM, for example through the production of 3D graphene-based scaffolds, has proved to be a novel strategy in directing the stem cell fate towards the osteogenic lineage. Nevertheless, a deeper understanding of the ability of graphene to improve the biological properties of different scaffold materials will be essential to engineer graphene-based substrates for targeted biomedical applications.

Regarding stem cells of dental origin, few data are available to date. Dental tissues are a very rich source of stem cells, which are collectively called Dental Stem Cells (DSCs) [9]. In the following paragraphs, studies describing applications of graphene-based nanomaterials in association with DSCs will be presented.

#### 2.2.1. Dental Pulp Stem Cells (DPSCs)

Dental pulp is the soft connective tissue of the tooth and Dental Pulp Stem Cells (DPSCs) are a unique MSCs population that is present in the cell rich zone and core of the pulp. These cells were firstly discovered in 2000 by Gronthos and colleagues, thanks to their ability to differentiate into odontoblasts/osteoblasts, adipocytes and neural-like cells [64].

Previous studies established that DPSCs and BM-MSCs are analogous cell populations, since they share expression profile for thousands of genes, among which those associated with the initiation of mineralization and bone homeostasis [65]. Other commonly expressed genes include those coding for various growth factors known to be strong promoters of osteogenesis, such as Fibroblast Growth Factor 2 (FGF-2) and Bone Morphogenetic Protein 2 (BMP-2) and BMP-4, but also genes encoding ECM components such as ALP, Collagen type I (COLI) and OCN. Human dental pulp is an optimal source of DPSCs due to its easy surgical access, the very low morbidity of the anatomical site after the collection of the pulp and the high efficiency of the extraction procedure of the stem cells from the tissue [66]. In addition, compared to equal volumes of bone marrow, dental pulp contains a higher number of MSCs.

**Figure 4:**
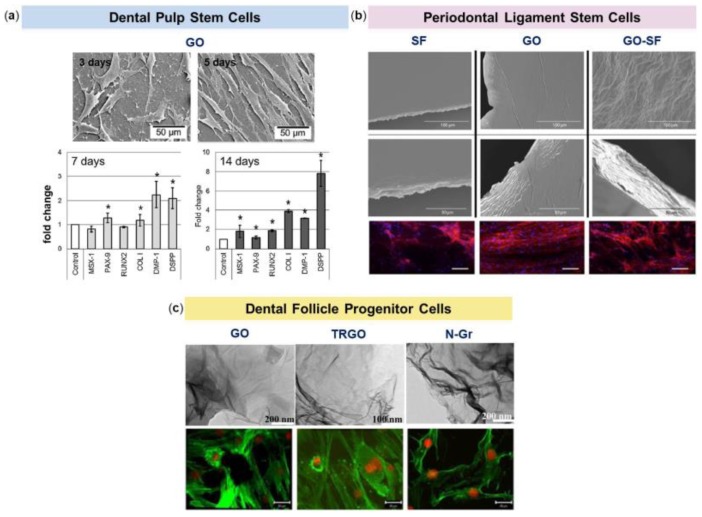
Interactions of graphene-based nanomaterials with Dental Stem Cells (DSCs). (**a**) SEM images showing that Dental Pulp Stem Cells (DPSCs) can efficiently adhere and proliferate on GO substrates for 3 and 5 days (top panel). DPSCs on GO present higher expression compared to glass (control) for all genes tested both at 7 and 14 days (bottom panel). (**b**) SEM images of films composed of Silk Fibroin (SF), GO and GO-SF mixture (3:1) at different magnifications (top panel). Immunofluorescence staining of the actin cytoskeleton showing a higher adhesion of Periodontal Ligament Stem Cells (PDLSCs) on GO and on the GO-SF composite film rather than on SF alone at 7 days. (**c**) TEM images of GO, Thermally Reduced Graphene Oxide (TRGO) and Nitrogen-doped graphene (N-Gr) (top panel). Confocal microscopy images of human Dental Follicle Progenitor Cells (DFPCs) seeded on GO, TRGO and N-Gr at 40 µg/mL showing staining of cytoskeleton actin filaments (green) and nuclei (red) (bottom panel). Reproduced with permissions from [67,79,89].

In the study of Rosa and coworkers, the effect of GO-based scaffolds on DPSCs proliferation and differentiation was evaluated and compared to that of glass substrates [67]. Cell morphology and proliferation were evaluated by SEM and fluorescence microscopy after 5 days from seeding; mRNA expression of Msh homeobox 1 (MSX-1), Paired box 9 (PAX-9), RUNX2, COLI, Dentin Matrix acidic Phosphoprotein 1 (DMP-1) and Dentin Sialophosphoprotein (DSPP) genes was measured at 7 and 14 days. The results of this study indicated that DPSCs were able to adhere on both the glass and GO surfaces, without any significant difference in proliferation. Nevertheless, when mRNA expression levels were measured, these resulted significantly higher for all genes tested onto GO compared to glass surfaces after 14 days from seeding (Figure 4a). MSX-1 is essential for the development of teeth, craniofacial structures and/or limb structures in embryos; it is a target activator of PAX-9 and the deletion of them causes tooth agenesis in mice [68,69]. RUNX2 is a transcription factor that is essential for osteogenic differentiation and can up-regulate the expression of OCN [70]. DMP-1 is highly expressed during odontogenesis and in mature odontoblasts, it is capable to produce dentin in vivo and its deficiency leads to dentinogenesis imperfecta [71]. DSPP codes for two proteins, dentin sialoprotein and dentin phosphoprotein, both of which have been associated with early dentinogenesis and are absent in bone [72]. What is really considered interesting in this work is the fact that the GO substrates provided an up-regulation for all the measured gene expression, even if no inducers for differentiation were used. In addition, the substrate was also capable of increasing the expression of both DMP-1 and DSPP, which are intimately related to odontogenic differentiation of stem cells from dental pulp. This means that the use of GO substrates by themselves may enhance the expression of odontogenic genes opening new opportunities to the use of GO alone or in combination with dental materials to improve their bioactivity and beyond.

In another study, the authors aimed at evaluating the potential of graphene to induce odontoblastic or osteogenic differentiation of DPSCs without the use of any chemical inducers [73]. Dentin and bone are considerably made of a HAp that is associated with the matrix produced by odontoblasts and osteoblasts, respectively [64]. Mineralization, odontogenic and osteogenic genes expression and protein expression of RUNX2, COLI and OCN were evaluated in DPSCs seeded on graphene and glass substrates for 14 and 28 days. Regarding the mineralization process, the graphene substrate positively influenced the cells to spontaneously secrete mineralized matrix. When comparing the gene expression levels, the odontoblastic-related genes MSX-1, PAX-6 and DMP-1 were significantly down-regulated on graphene substrates compared to glass. On the contrary, graphene increased both gene and protein expressions of RUNX2 and OCN, thus suggesting its ability to induce osteogenic differentiation rather than odontogenic differentiation of DPSCs. An explanation to this may be the high Young modulus of graphene (1.0–2.4 TPa) [74], which can contribute to the osteogenic differentiation of DPSCs as these cells usually need soft substrates to achieve the odontogenic differentiation [75]. Taken together, the results of this study suggest that DPSCs are not prone to differentiate into odontoblastic-like cells when grown on graphene, but this substrate is able to induce osteogenic differentiation of the cells without the use of chemical inducers for osteogenesis.

Therefore, authors seem to agree with the fact that graphene is a biocompatible material for DPSCs and it might be used as a valid substrate for dental tissue engineering since it is able to promote either osteogenic or odontogenic differentiation of DPSCs depending on the surface characteristics and functionalization of graphene, as also emerged in the previous section.

#### 2.2.2. Periodontal Ligament Stem Cells (PDLSCs)

The periodontium refers to those structures which surround and support teeth. It is composed by four parts: gingiva, alveolar bone, cementum and periodontal ligament. The function of the periodontal ligament is to literally attach the teeth to the bone and when a chronic process, called periodontitis, affects these structures, the teeth stability and health are compromised. All the periodontium is constantly maintained by Periodontal Ligament Stem Cells (PDLSCs), which have the ability to differentiate into cementoblasts, odontoblasts and fibroblasts [76].

Thanks to their potential to differentiate into several types of specialized cells, PDLSCs can be used as substitute to BM-MSCs in order to determine the osteogenic potential and the viability onto different substrates [77]. Even if in literature the bone formation potential of PDLSCs on various surfaces such as Titanium (Ti) has been already demonstrated [78], few articles evaluated the biocompatibility of graphene-based nanomaterials for human PDLSCs.

In one of the few studies, the effects of GO, Silk Fibroin (SF) and GO combined with SF were investigated on PDLSCs adhesion, proliferation, viability and expression of MSCs markers [79]. SF is widely used as a biocompatible material in the fabrication of cellular scaffolds for tissue engineering and a composite of the two materials has already been proposed for several biomedical applications [80,81]. Nevertheless, its performance as a substrate for growing of PDLSCs has not been addressed yet. In this work, PDLSCs obtained from healthy extracted molars were used and cultured up to 10 days on the above-mentioned substrates and on plastic, which represented the control condition. Cell adhesion was higher on GO and on the GO-SF composite film rather than on fibroin alone, as revealed by immunofluorescence staining of the actin cytoskeleton (Figure 4b). Regarding the cells proliferation rate, the results of the MTT assay showed a high rate of proliferation for PDLSCs growing on GO films compared to plastic and on GO and fibroin composite film compared to fibroin alone after 10 days from seeding. In order to evaluate if the biomaterials employed in this study were able to maintain the mesenchymal phenotype of PDLSCs, flow cytometry was used to assess the expression of some MSCs surface molecules. Culture of PDLSCs on SF, GO or GO-SF composite did not significantly alter the level of expression of the typical MSCs markers CD73, CD90 or CD105 compared to expression levels displayed by PDLSCs cultured on plastic The authors concluded that the GO-SF composite significantly improved the performance of the fibroin film, as an alternative to the coating with collagen, representing an interesting combination of biocompatibility, induction of proliferation and mechanical resistance, very suitable for working in cellular environments where mechanical resistance is required.

Later, the same group investigated the potential of SF and GO composites to promote human PDLSCs spontaneous differentiation into osteo/cementoblast-like cells [82]. In the study, the authors have optimized the parameters of fabrication of the GO-SF composite film, using different ratios of the two materials and also varying the graphene oxidation status, with the aim to find the best configuration for cell proliferation and differentiation. The PDLSCs proliferation rate was consistently improved in those combinations containing low amounts of graphene and a high SF dose, as emerged from the MTT assay results at 7 and 10 days of culture. The authors concluded that the best configurations in terms of PDLSCs proliferation were GO alone and rGO:rSF at 1:3 ratio. SF was used to confer 3D characteristics to the GO or rGO as well as to improve its handling. Previous data have shown that human PDLSCs bioengineered on 3D graphene scaffold preparations are associated with higher proliferation rates than on 2D ones [83]. Flow cytometry analysis was further used to confirm the mesenchymal phenotype of the isolated PDLSCs and to determine possible phenotypic changes after their culture on the different graphene-fibroin combinations. The MSCs surface molecules CD73, CD90 and CD105 were found to be present in the PDLSCs grown on all the biomaterials tested, although their expression decreased with culturing time. This is not surprisingly, since the expression level of MSCs markers progressively declines in stem cells during their multilineage differentiation process [84,85]. After demonstrating the beneficial effects of GO-SF composites on PDLSCs proliferation, gene expression analyses were performed to further characterize the effect of these scaffolds on PDLSCs differentiation into osteo/cementoblast-like cells. No osteogenic chemical inducers were added to the culture medium. GO-SF composites, particularly the reduced configurations rGO, rSF and rGO-rSF, were found to induce the over-expression of early osteoblast/cementoblast markers, including BMP2, RUNX2, ALP and COLI. On the contrary, Osterix (OSX) and Osteocalcin (OCN), whose expressions are associated with late osteoblast differentiation stages, were found to be down-regulated on all the substrate preparations. Starting from this result, the authors explored the expression of two specific cementum-related genes, Cementum Attachment Protein (CAP) and Cementum Protein 1 (CEMP1), that are expressed at early and late differentiation stages, respectively [86]. All the scaffolds tested were associated with significant down-regulation of CAP and concomitant over-expression of CEMP1, suggesting that graphene-fibroin composites can induce cementoblast differentiation of the human PDLSCs in the absence of any growth factors.

#### 2.2.3. Dental Follicle Progenitor Cells (DFPCs)

The dental follicle is the loose connective tissue surrounding the enamel organ and the dental papilla of the developing tooth germ. Its principal role is the coordination of tooth eruption through the regulation of the osteoclastogenesis and osteogenesis processes [87]. The Dental Follicle Progenitor Cells (DFPCs) are multipotent stem cells that have immunomodulatory properties, high proliferation rate and ability to differentiate into odontoblasts, cementoblasts, osteoblasts and other cells implicated in the teeth [88]. Furthermore, they are able to re-create a new periodontal ligament after in vivo implantation.

To date, only one study evaluating the behavior of human DFSCs on graphene-based nanomaterials has been reported [89]. In the work, human DFSCs obtained from healthy extracted teeth were seeded onto GO, Thermally Reduced Graphene Oxide (TRGO) and Nitrogen-doped graphene (N-Gr) substrates. Then, cytotoxicity, oxidative stress induction, cellular and mitochondrial membrane alterations were analyzed for all the developed substrates (Figure 4c). The results of this study showed that GO was the less toxic for the cells, followed by N-Gr, whereas the TRGO substrate resulted the more toxic. Regarding the oxidative stress level, both the GO and the TRGO substrates induced lipid peroxidation without significant alteration of the membrane. On the other hand, the N-Gr substrate, which showed to have a positive antioxidant effect on the stem cells studied, reduced cell viability without oxidative stress. The authors suggested that N-Gr affected the cells not only determining a physical damage of the plasma membrane due to graphene irregularities, but also by the ability of nitrogen to coordinate biomolecules thus interfering in the biological processes. As regards the mitochondria membrane potential, it was decreased in all the substrates used in this study following a dose-dependent manner. This effect was higher in the TRGO than in the GO substrate, while in the N-Gr substrate structural lesions were present only at high concentrations (20 and 40 µg/mL). The authors concluded that while GO and N-Gr may be considered as promising fillers for various dental nanocomposites, TRGO does not seem to be a suitable material.

### 2.3. Graphene and Immune Cells

A critical step for the application of nanotechnology in medicine is the interaction of a material with the immune system because, once administrated, it immediately enters in contact with the immune cells. Graphene and its composites are not exempt from the interaction with cells of the immune system and, as in the last few years the biological applications of graphene-based nanomaterials have grown, it results necessary to investigate the immune-related impact of these nanomaterials. Many papers about this issue underlined stimulation or suppression effects of graphene-based nanomaterials on immune system cells, depending mainly to graphene purity, oxidation level, shape dimension, functionalization and to the type of immune cells analyzed.

The first extended investigation on graphene and immune cells was made by Sasidharan and coworkers [37]. After comparing the effects of pristine graphene and GO on Peripheral Blood Mononuclear Cells (PBMCs), the authors observed really different effects: pristine graphene induced a significant release of Interleukin-8 (IL-8) and IL-6 whereas GO determined only a moderate release of IL-8, despite both seemed to have an excellent compatibility with PBMCs.

To best characterize the impact of GO on immune cells, Orecchioni and colleagues studied and compared the effects of small-size (100–500 nm) and large-size (1–10 µm) GO flakes on a pool of PBMCs [90]. The authors detected a more efficient cellular activation and cytokine release by small-size GO. In particular, through gene expression analysis, they underlined that small GO is able to modulate a large number of genes related to cytokines and activation-related genes, such as T-box transcription factor (TBX21) and CD80. Furthermore, it was found that GO promotes the over-expression of many pathways such as leukocytes chemotaxis pathway and genes associated with C-X-C motif chemokine 10 (CXCL10) ligand and C-X-C motif chemokine receptor 3 (CXCR3), able to activate T-helper and Natural Killer (NK) cells. Once established various effects on the total PBMCs, several studies have then been performed also analyzing the effect of graphene on specific immune cell types, as described in the following paragraphs.

#### 2.3.1. Lymphocytes

Lymphocytes, which represent about the 20–40% of the white blood cells and the 70–80% of PBMCs, are responsible for the antigen-specific and innate immune response. The most of research have been made analyzing the effects of graphene-based nanomaterials on T lymphocytes, concluding that the cytotoxicity of graphene depends on its dosage, time of exposure and type of material with which these are combined. For example, Ding and coworkers demonstrated that low doses of GO have no cytotoxic effects on T lymphocytes, while a dose over 100 µg/mL causes oxidative stress related apoptosis after 24 h of incubation [91]. In particular, at high concentration GO was found absorbed on cell membrane, without internalization or cell membrane disruption, but it caused a reduction of cell viability and an increase of lymphocytes apoptosis probably due to the production of ROS. However, no significant reduction in T lymphocytes immune response was found. The same tests conducted on GO-polyethylenimine (GO-PEI) revealed that this kind of functionalization severely damaged T lymphocytes and suppressed their immune ability [91]. On the contrary, the functionalization of GO with polyvinylpyrrolidone (PVP) increased the biocompatibility of GO and at high doses of GO-PVP the amount of apoptosis was much less then in GO alone [92].

#### 2.3.2. Macrophages

Half of the studies about graphene and the immune system have been carried out on macrophages, not only because these represent the first phase of body immune reaction, but also because macrophages are the easiest long-term culturing primary immune cell population [93].

**Figure 5:**
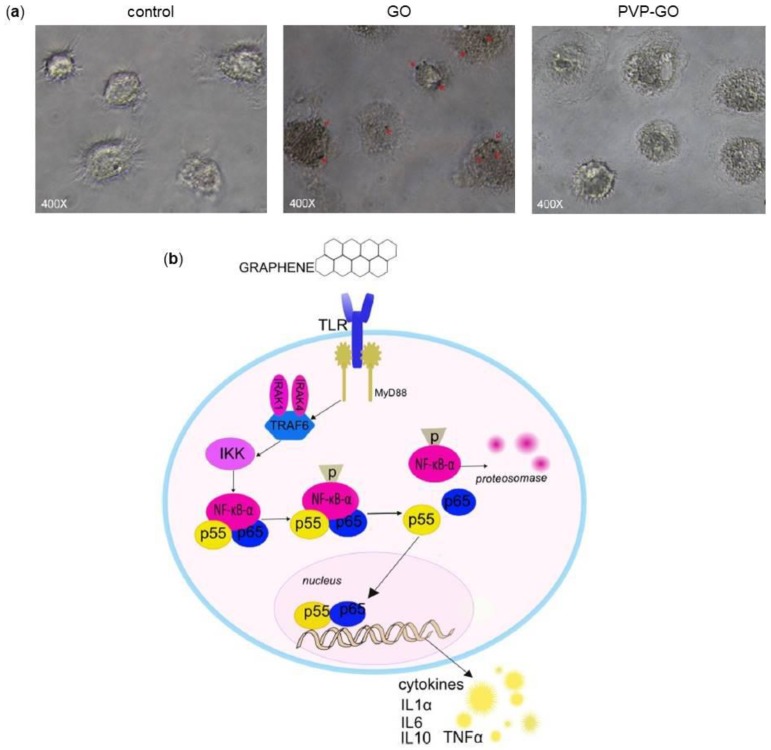
Interactions of graphene with macrophages. (**a**) Optical micrographs of macrophages treated with 25 µg/mL GO and PVP-GO for 48 h. Macrophages showed to be inclined to GO internalization (red arrows), while the functionalization with PVP prevents the phenomenon. (**b**) Signaling pathway of macrophage activation stimulated by graphene. Graphene may stimulate Toll-Like Receptors (TLRs), thus activating kinase cascade Myeloid Differentiation primary response gene 88 (MyD88)-dependent mechanism. IKK activation initiates the phosphorylation and degradation of IκB and consequently, the release of NF-κB subunits and their translocation into the nucleus. NF-κB binds to the promoter regions of its effector genes and initiates the transcription of multiple pro-inflammatory genes and the secretion of Interleukin 1α (IL-1α), IL-6, IL-10, Tumor Necrosis Factor alpha (TNF-α). Reproduced with permissions from [92,99].

The phagocytic activity of macrophages makes them the most exposed cells to the negative influence of graphene and its derivatives [94]. It has been reported that the ability of macrophages to the internalization mainly depends on the size of the sheets: the smaller is the lateral dimension of GO, the greater the ability of macrophages to internalize it [95]. Moreover, the capacity of macrophages to phagocyte GO can be modulated by GO functionalization. Zhi and colleagues demonstrated that the functionalization of GO with PVP decreases the internalization of the compound, indicating that PVP-GO has a better immunological biocompatibility (Figure 5a) [92].

Regarding the activation of macrophages, Chen and colleagues showed that pristine graphene and GO promoted the production of several cytokines, such as IL-1α, IL-6, IL-10 and Tumor Necrosis Factor alpha (TNF-α), via NF-κB pathway, that is activated via Toll-Like Receptors (TLRs) [96]. Graphene promotes the activation of IκB Kinase (IKK), thereby activating NF-κB [97,98]. Moreover, GO stimulates the production of Myeloid Differentiation primary response gene 88 (MyD88), another protein related to TLRs. Activated TLRs start the kinase cascade, by a MyD88 mechanism, that promotes the translocation of NF-κB into the nucleus, thus increasing cytokines gene expression (Figure 5b). In addition, an elevated activation of TLRs, especially TLR-4, causes TLR-dependent necrosis of macrophages. The silencing of genes that code for TLRs, completely immunize macrophages from graphene [99]. The stimulation of TLRs also favors the formation of phagosomes that active enhance the uptake of graphene and the consequent affection of cell metabolism and gene/protein expression [99]. Experiments on phagocytic process showed that 100 µg/mL GO generate the same effects of LPS stimulation that is the activation of macrophages and the production of pro inflammatory cytokine [100].

#### 2.3.3. Dendritic Cells (DCs)

Dendritc cells (DCs), representing the 1–2% of PBMCs, play a fundamental role in the activation of antigen-specific T lymphocytes [101]. Few data are available about interactions of graphene-based nanomaterials and DCs. Tkach and coworkers showed that GO incorporated by DCs down-regulates the intracellular level of LMP7, a part of an immunoproteasome required for processing protein antigen [102]. In another study, Zhi and colleagues compared the effect of GO and PVP-GO on DCs, highlighting a capacity of GO to promote maturation of DCs and also the overexpression of IL-6 in a dose-dependent manner, compared to the non-treated group [92]. The authors concluded that functionalization with PVP mitigate the effects of GO, that resulting in a lower immunogenicity.

### 2.4. Graphene and Antibacterial Activity

Infection is a frequent process during biomaterial implantation procedures. Since antibiotics often impact negatively on bacterial flora and pathogens are able to acquire resistance against different antibiotics, in the last few years various nanotechnologies with antimicrobial properties have been developed and studied [103,104], trying to get closer to the ideal scaffold that should inhibit bacterial growth at the surface, while simultaneously promoting cell adhesion and proliferation [43].

It has been reported that some graphene-based nanomaterials possess antibacterial properties; nevertheless, the effects of graphene on bacteria structure, metabolism and viability depend on the materials’ concentration, time of exposure, physical-chemical properties, as well as on the characteristics of bacteria used in the tests [105,106].

A lot of studies indicated that graphene and several graphene nanocomposites have a remarkable antibacterial ability against both Gram-negative and Gram-positive bacteria [107,108,109,110]. It has been demonstrated that the antibacterial effect is due to the capacity of graphene to physically damage microorganisms through different mechanisms: graphene nanostructure acts as a nano-knife penetrating and cutting cell membrane, it wraps cells inducing mechanical stress, it is able to extract phospholipids from lipid membranes and it produces oxidative stress through ROS generation but also by charge transfer phenomena. Molecular dynamic simulations suggest that thin graphene nanosheets can insert into both bacterial membranes creating a break and, once inserted, the Van Der Walls forces and the hydrophobic properties of graphene promote phospholipids extraction from the lipid layers of the bacterial membranes causing irreversible damages [111].

Another way for graphene to exert antibacterial activity seems to be through the separation of microorganisms from the microenvironment. Aggregated graphene sheets in suspension can isolate bacteria from the surrounding environment hindering nutrient consumption, reducing their ability to proliferate and favoring their inactivation [108].

Graphene antibacterial activity is also probably related to oxidative stress induction. This is mainly mediated by ROS generation, especially when GO is used, nevertheless oxidative stress can be triggered without ROS generation. Li and coworkers showed that graphene is able to act as an electron pump pulling out electrons from bacterial membranes and inducing ROS-independent oxidative stress that affects microorganisms [110].

Obviously, these mechanisms are not specific against bacteria but can also affect other cells, although with less effectiveness. Pang and colleagues tried to clarify the ratio between biosafety and antibacterial activity of graphene and GO [112]. They observed that both cytotoxicity and antibacterial effects are dose-dependent and that GO has a higher activity; in particular, a GO concentration in the range of 50–100 µg/mL keeps the balance between minor cytotoxic effects and major antibacterial activity.

**Figure 6:**
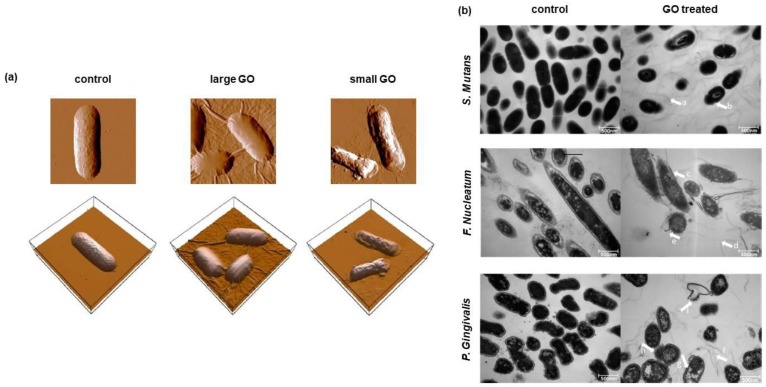
Effect of GO nanosheets on bacteria. (**a**) Atomic Force Microscopy (AFM) amplitude (top) and 3D (bottom) images of *Escherichia coli* cells 2 h of after incubation with/without GO sheets. *E. coli* cells incubated with deionized water without GO sheets show a preserved integrity of the membrane (control). The incubation with the 40 μg/mL large GO sheets suspension results in a completely cover of bacterium surface by GO sheets, whereas small GO sheets adhere to cell surface without fully covering it. Scale bars are 1 μm. (**b**) TEM images of *Streptococcus mutans, Fusobacterium nucleatum* and *Porphyromonas gingivalis* cells after incubation with GO nanosheets dispersion (right side) for 2 h and after incubation with saline solution for 2 h as control (left side). All treated cases had the same GO dose of 80 μg/mL. Scale bars are 500 nm. Reproduced with permissions from [113,114].

Among graphene derivatives, GO attracted particular attention thanks to the ease with which it can be functionalized, representing the precursor of the most graphene nanocomposites, but also thanks to its excellent water dispersity. However, a lot of study established that antibacterial activity of GO is dependent not only on its concentration but also on the size and the lateral dimension of GO sheets. Liu and colleagues demonstrated that the antibacterial activity of GO nanosheets depends on their size [113]. Indeed, larger GO sheets express stronger antibacterial activity against *Escherichia coli* compared to smaller one, probably due to the capacity of larger GO sheets to completely cover bacteria inhibiting their proliferation and colony formation (Figure 6a).

In the environment of the oral cavity, *Streptococcus mutans*, *Porphyromonas gingivalis* and *Fusobacterium nucleatum* are the most representative bacteria responsible for caries, periodontal and periapical diseases [114]. The microbial community resident in the mouth exists in balance with the oral microenvironment [115]. Host susceptibility, diet and habits could lead to a break in balance that gives rise to adverse reactions. In particular, *S. mutans* is a Gram-positive facultative anaerobic bacterium importantly involved in caries formation and in the modification of the oral microenvironment, decreasing the pH value by the production of large amount of organic acids [116]. Instead, *P. gingivalis* and *F. nucleatum* are Gram-negative anaerobic bacteria, associated with periodontitis [117]. For these reasons, antibacterial activity of graphene and its nanocomposites, in particular against these cariogenic bacteria, has been studied. He and collaborators investigated the effect of GO against dental pathogen bacteria, showing that the viability of *S. mutans*, *P. gingivalis* and *F. nucleatum* decreased in the presence of GO nanosheets depending on its concentration in a dose-dependent manner [114]. TEM images clearly showed that, when the GO was present, the integrity of *S. mutans*, *P. gingivalis* and *F. nucleatum* was strongly compromised due to the severe insertion, cutting and destructive extraction of lipid molecules effect that GO act against the membrane (Figure 6b).

Graphene and some of its composites seem to exert their activity not only against single bacteria but also against bacterial biofilms. Biofilms are surfaced-attached bacterial communities that self-produce adhesive ECM; they play a role in a wide variety of infections, i.e., caries, catheter infection and bloodstream infection [118]. Several complicated and expensive methods to prevent biofilms formation have been proposed, including coating of nanomaterials with ion or polymers impregnated with antibiotics [119,120,121,122]. Recently, inspired by graphene antibacterial ability and its ease functionalization, possible effects of graphene-based nanomaterials against biofilms are being studied. For example, Song and coworkers investigated the influence of GO on bacterial biofilm formation, observing that high GO concentrations inhibit the formation of Gram-negative and Gram-positive biofilms via membrane stress, whereas low GO concentrations enhance their formation [123]. The authors hypothesized that low GO concentration kills only a limited part of bacteria and dead cells could serve as a protection barrier and nutrient to the rest of biofilm formation, whereas high GO concentration promotes the inactivation of most bacteria, hindering the biofilm growth. In another work, Mao and colleagues tested the antibiofilm activity of a GO-aptamer composite and compared to that of GO and aptamers per se [124]. They showed that all agents interacted with pathogen disturbing the initial growth of biofilm and destroying the established biofilm, but the combination of GO and aptamers exhibited a superior synergic effect than the single substances.

Despite a lot of studies stressed an antimicrobial activity for pristine graphene and GO, some other works evidenced that graphene has no intrinsic bactericidal properties, but even it is able to enhance bacteria growth when a colloidal suspension is formed [125,126]. Das and Ruiz in their studies showed that only the functionalization with antibacterial agents such as silver nanoparticles conferred to the graphene-based nanomaterials a bactericidal activity [125,126]. In agreement with these studies, Some and collaborators confirmed that graphene alone has no antibacterial properties, but graphene-iodide composites, above all double-oxidizes GO-iodide, have a potent antibacterial activity [127].

Wu and coworkers tried to clarify the debate concerning the antimicrobial activity of graphene and its derivatives comparing the antibacterial effects of GO, GO-polyoxyalkyleneamine (GO-POAA) and GO-chitosan against *E. coli* and *Bacillus subtilis*, Gram-negative and Gram-positive bacteria, respectively [128]. The results of this study indicated that less than 50 µg/mL GO in a nutrient medium solution has no antimicrobial activity; on the contrary, it enhances bacterial growth acting as a biofilm that allows bacterial attachment and proliferation. On the other hand, the conjugation of GO with POAA or chitosan, two antibacterial molecules, at the same concentration showed antibacterial effects. All materials demonstrated antibacterial activity when bacteria were grown in Phosphate Buffered Saline (PBS) solution.

All these studies would suggest that the antibacterial activity of graphene-based nanomaterials is variable and dependent on the conditions of the tests; in particular, results change depending on the type of material and the size of particles, on its concentration and its state (in solution or adsorbed on a support), on the type of bacteria used in the study and on the medium used for their growth.

## 3. Applications of Graphene-Based Nanomaterials in the Dental Field

Graphene and its derivatives display many potential applications in the dental field, as can be seen from the literature. Thanks to their potentiality, particularly in driving the osteogenic differentiation of stem cells and antibacterial abilities, the application of graphene-based nanomaterials to already existing dental technologies are being studied. In addition, graphene seems to be interesting as a platform able to release therapeutic molecules to improve implants osseointegration and bone formation. In the following paragraphs, we will present several studies discussing the association of graphene with dental implants, membranes, resins, cements and adhesives, as well as strategies for teeth-whitening.

### 3.1. Graphene and Dental Implants

Nowadays, Ti dental implants are considered as the best substitutes for missing teeth thanks to their reliability and predictability, besides to their mechanical strength and favorable biocompatibility [129,130]. Since these devices are in close contact with the surrounding tissues, a critical parameter for the success of the implantation is the ability of the implant to integrate with the tissue promoting the osseointegration process; this represents indeed a fundamental step for obtaining the best new bone quantity and quality [129,131]. During this process, a key role is played by the implant surface characteristics such as roughness, surface treatment and hydrophilicity.

Ti inertness represents a disadvantage because it may induce the development of fibrous tissue which could lead to the failure of the implant [130]. For this reason, several studies have focused on modifying implant surfaces to favor a better osseointegration. In this context, graphene appeared to be an excellent implant-coating for hard tissue engineering in order to accelerate bone regeneration.

As evidenced earlier, GO-coating was found to be a successful substrate for anchoring and growing of DPSCs on its rough surface and for promoting their osteogenic differentiation through up-regulation of MSX-1, PAX-9, RUNX2, COLI, DMP-1 and DPSS in particular after 14 days of culture [67]. To improve stem cells osteogenic differentiation, the groups of Jung and Ren functionalized GO-Ti implants using different methods with dexamethasone, a synthetic glucocorticoid that is known to contribute to this phenomenon [130,132]. Independently of the method of functionalization and coating, both groups showed that GO-coating of Ti implants, even more when functionalized with dexamethasone, improves implants biocompatibility, cell proliferation and mostly cell osteogenic differentiation.

**Figure 7:**
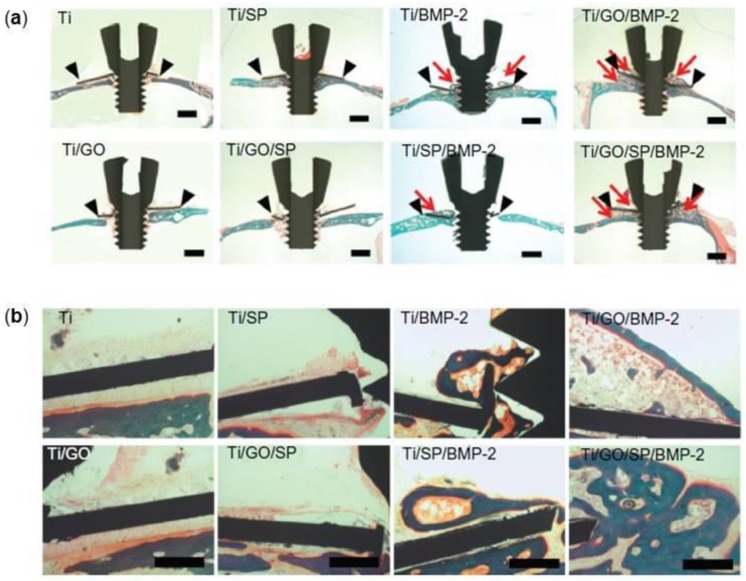
Bone regeneration of Ti implants with or without GO coating and BMP-2/SP loading in mouse calvarial defects 8 weeks after treatment. The red arrowheads indicate the newly formed bone, the black arrowheads indicate the implant at (**a**) 12.5× magnification and (**b**) 100× magnification. Reproduced with permissions from [136].

Another approach to improve the osseointegration of Ti implants could be its coating with bioactive proteins. For bone regeneration, BMP are the most potent osteoinductive proteins and among these, BMP-2 has been reported to induce osteogenic differentiation of stem cells, which can enhance the osseointegration of implants by forming bone at the space between the implants and the implantation site [133,134]. La and collaborators investigated the efficiency of a Ti substrate coated with GO as a delivery carrier for BMP-2, an osteoinductive protein that need to be released over a long time period and Substance P (SP), a stem cells recruiter protein [135], for bone regeneration [136]. In vitro experiments showed that no significant difference was found in SP release between Ti and Ti/GO. Interestingly, the BMP-2 release from the Ti/GO substrate was maintained for 14 days; on the contrary, the release of almost all the BMP-2 content from the Ti substrate occurred on the first day. Furthermore, the bioactivity of the BMP-2 released from the substrate was significantly higher in the GO-coated group. The in vivo study, in which the different substrates were implanted on mouse calvaria, showed that even if no significant difference in bone formation was found between Ti/BMP-2 and Ti/SP/BMP-2, Ti/GO/SP/BMP-2 implants showed much more extensive bone formation than Ti/GO/BMP-2 implants confirming that the presence of GO preserves the bioactivity of proteins (Figure 7).

Implant GO-coating represents an advantage also due to its antibacterial properties. Despite the fixation of graphene on Ti implant makes it to lose a bit of antibacterial activity, there is the possibility to functionalize the coating with antibacterial substances, such as antibiotics or silver nanoparticles. As demonstrated by Quian and colleagues, minocycline hydrochloride added to GO-coating improved the antibacterial activity against aerobic or facultative anaerobic bacteria (*Staphylococcus aureus*), facultative anaerobic bacteria (*E. coli*) and anaerobic bacteria (*S. mutans*) thanks to the synergic effect of GO contact-killing and minocycline release-killing [137]. In another study, the group of Jianfeng tested the antimicrobial activity of GO-silver coating on Ti, showing that it is very prominent against *S. mutans* and *P. gingivalis* [138]. This suggests that this multiphase nanocomposite could be helpful in the prevention of implant-associated infection.

As previously reported, human PDLSCs are promising DSCs that can be used as an alternative to human BM-MSCs and GO is believed to be a useful platform for modulating structure and function of these cells. Nevertheless, the behavior of PDLSCs on GO-coated Ti substrates is not fully understood. In the work of Zhou and colleagues, the morphology, proliferation and osteogenic differentiation potential of PDLSCs seeded on GO-Ti scaffolds were evaluated and compared to those obtained on sodium titanate (Na-Ti) substrates [139]. The results of this study showed that, when the cells were seeded onto GO-coated Ti scaffolds, they exhibited higher proliferation rate, ALP activity and up-regulation of gene expression levels of the osteogenesis-related markers COLI, ALP, Bone Sialoprotein (BSP), RUNX2 and OCN respect to those cultured on the Na-Ti substrate. In addition, GO enhanced the expression of RUNX2, BSP and OCN also at a protein level. The authors concluded that the combination of GO, PDLSCs and Na-Ti can represent a big step forward in regenerative dentistry. Nevertheless, further additional studies will be necessary to elucidate how to use GO as a biocompatible and implantable platform for the delivery of therapeutic proteins for applications related to regenerative medicine, especially for the success of Ti dental implants.

### 3.2. Graphene and Membranes

To increase efficiency of bone repair, especially in periodontal and periimplant bone defects, the use of Guided Bone Regeneration (GBR) membranes has been important for years in oral surgery. These membranes are placed on the bone defect regeneration site and their principal function is to not allow infiltration of soft tissue cells into the growing bone. Thus, these act as physical barriers that separate connective tissue and the regenerating bone providing the slow osteogenic cells migration [140]. In the dental field, the use of membrane for GBR was introduced in 1980’ to stop cell migration from gingival connective tissue and epithelium to periodontal defect [141]. In the last decades, several types of membrane, non-resorbable and bioabsorbable, have been developed and tested for the application in GBR (Table 1) [142].

**Figure 8:**
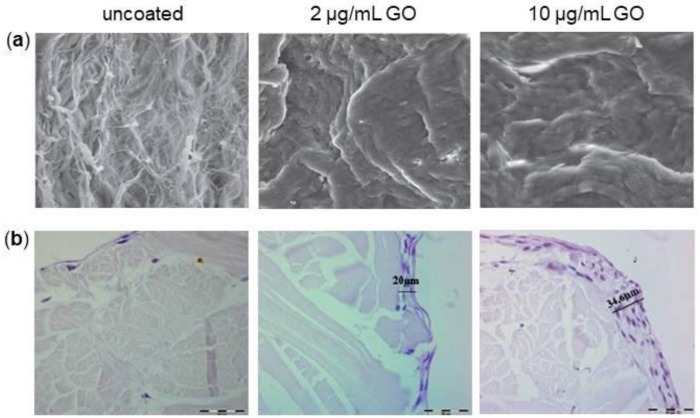
Different GO coating concentration on collagen membrane from porcine dermis. (**a**) SEM images of uncoated, 2 μg/mL and 10 μg/mL GO-coated membranes. 4.05 k magnification. (**b**) Hematoxylin-Eosin staining of uncoated, 2 μg/mL and 10 μg/mL GO-coated membranes with DPSCs after 28 days of culture. 40× magnification. Reproduced with permissions from [144,145].

All membranes need to satisfy the five main criteria underlined by Scantlebury: biocompatibility, space-making, cell-occlusiveness, tissue integration and clinical manageability [143]. In order to improve these main criteria, graphene started recently to be used in this field. For example, De Marco and colleagues enriched collagen membranes with two different concentration of GO (2 µg/mL and 10 µg/mL) and tested the effect on Human Gingival Fibroblasts (HGFs) [144]. They showed that the presence of GO on collagen membranes altered membrane features: it conferred a lower deformability, higher stiffness and reduced hydration and increased roughness compared to non-coated membranes. These changes favored the proliferation of HGFs, avoiding any inflammatory response, as demonstrated by the reduction of IL-6 and Prostaglandin E2 (PGE2) secretion after 3 days of culture and facilitated proteins adhesion to the membrane (Figure 8a).

The same GO-coating applied on collagen membranes was tested using DPSCs to analyze osteoblastic differentiation and inflammatory response [145]. The researchers found that GO-coating significantly increased cell viability on a concentration-dependent manner and promoted DPSCs osteoblastic differentiation, as demonstrated by the up-regulation of BMP-2, RUNX2 and OSX gene expression. Moreover, 10 μg/mL GO enrichment reduced the expression of TNF-α at all experimental times compared to the control membrane, whereas 2 μg/mL GO enrichment significantly decreased TNF-α expression only after 21 days of culture. Interestingly, Hematoxylin-Eosin staining revealed that cells were not able to penetrate into the membrane and the more concentrated GO coating led to the formation of a thicker cell layer (Figure 8b). The authors concluded that the GO enrichment of collagen membranes promoted osteoblastic differentiation process, decreased inflammation and was compatible with cell viability in a dose-dependent manner. These new membranes could be considered as a valid alternative to substitute conventional collagen membranes.

### 3.3. Graphene and Resins, Cements and Adhesives

Resins, cements and adhesives are the most used materials for dental restoration. Nevertheless, their porosity and adhesiveness make them receptacles for bacteria in the oral cavity and in particular in proximity of dental restoration. These polymeric materials, indeed, facilitate the adhesion of bacteria and the formation of biofilms that are the main cause of dental restoration failure [146]. An example of the application of graphene to dental commercial materials is reported in the study of Bregnocchi and coworkers [146]. The authors added Graphene Nanoplatelets (GNPs) as nanofiller to a commercial dental adhesive. The generated graphene nanocomposite significantly inhibited the growth of *S. mutans*, without altering the standard adhesion properties of the dental adhesive.

One of the main limitation of GNPs in dental application is its grey color. In order to ameliorate this aspect, Zanni and colleagues tested a hybrid material composed by Zinc Oxide Nanorods (ZnO-NRs) grown on GNPs, thus combining the antimicrobial effect of GNPs with the light color and biocidal properties of ZnO-NRs [147]. The authors studied the effect of Zinc Oxide Nanorods-Decorated Graphene Nanoplatelets (ZNGs) against *S. mutans*. The results showed that up to the 95% *S. mutans* cell viability reduction was obtained using ZNGs even at a very low concentration (5 μg/mL). Moreover, the biomass and exopolysaccharide production, which are necessary for the biofilm formation, were evaluated: the ZNGs showed to behave as an obstacle for the biofilm growth. The authors concluded that the use of ZNGs is a viable instrument to control caries disease as it decreases *S. mutans* growth.

Biris and colleagues successfully prepared graphene sheets embedded with various amounts of gold nanoparticles (Gr-Au-x) using the radiofrequency catalytic chemical vapor deposition technique over a Au_x_/MgO catalyst [148]. Sarosi and colleagues analyze the effect of these sheets used as nanofiller for some dental nanocomposites based on BisGMA/triethyleneglicol dimethacrylate matrix [149]. The authors concluded that the graphene–gold nanoparticles could be very promising filler for the dental nanocomposites because the reinforcement with high percent of nanoparticles is a good solution to improve physicochemical properties.

As a last example, Li and collaborators demonstrated that the functionalization of glass ionomer cements with fluorinated graphene is not only useful to inhibit bacterial growth but also to ameliorate mechanical properties of the cements, increasing microhardness and compressive strength and decreasing friction coefficient, all important parameters for cements [150].

### 3.4. Graphene and Teeth-Whitening

Nowadays, a lot of people would change the color of their teeth, which often is ruined by food, beverage and smoke; consequently, the level of demand for tooth-whitening is increasing. Tooth color is determined by intrinsic factors, associated with the light scattering and absorption properties of the enamel and dentine and extrinsic factors, associated with the absorption of materials (e.g., tea, red wine) on the surface of the enamel.

The products used for tooth-whitening are extensively-based upon Hydrogen Peroxide (H_2_O_2_) chemistry, because of its bleach-properties and the techniques constantly evolve to ameliorate the rate of whitening, the ease of the treatment and to decrease the side effects. The most common risks of teeth-whitening include increase dental sensitivity and gingival irritation, besides changes in tooth microstructure. These effects are directly related to the concentration of H_2_O_2_, the duration of the treatment and the non-bleach component of the products [151].

**Figure 9:**
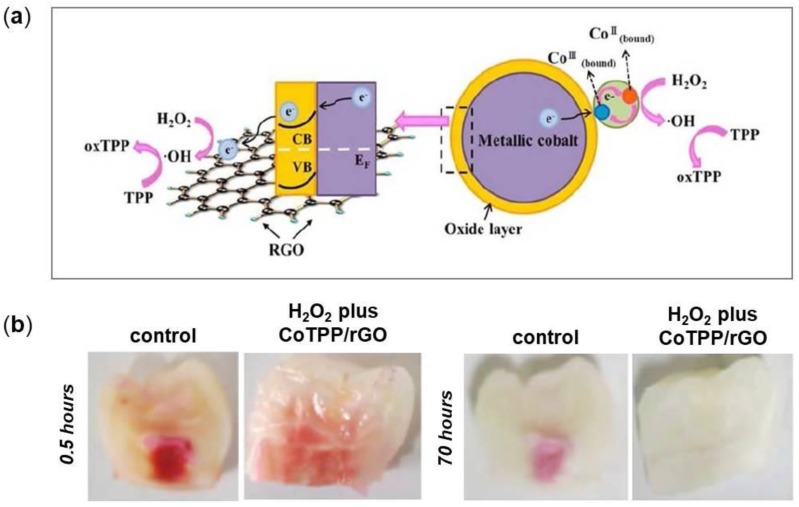
Strategies based on graphene for improving teeth-whitening. (**a**) A schematic diagram illustrating the enhanced peroxidase-like catalytic activity of the rGO-Co. Reactions of Cobalt Tetraphenylporphyrin (CoTPP) with Hydrogen Peroxide (H_2_O_2_): Co_III_ TPP–e_2_1(1/2) H_2_O_2_ → (Co_IV_) TPP-OH_2_ (Co_IV_) TPP-OH → 2 Co_III_ TPP1O_2_12H_1_. (**b**) Photographs of teeth stained with dye D&C Red 34 and bleached using H_2_O_2_ alone or H_2_O_2_ plus CoTPP/RGO for 0.5 (left) or 70 h (right). Reproduced with permissions of [150].

To improve the bleaching process and decrease the time of the treatment, the light-activation of peroxide-based products is used, but novel ways are being found to ameliorate the efficiency of teeth-whitening. Several studies reported that the use of activating agents, such as nanoHAp or Fe(III) phthalocyanine, together with H_2_O_2_ is useful for the purpose [152]. Based on these evidences, Su and coworkers proposed the application of a nanocomposite made of rGO and Cobalt Tetraphenylporphyrin (CoTPP) as a catalyst for tooth bleaching (Figure 9a) [120]. They then evaluated its efficacy on tooth-whitening compared to the whitelight irradiation and D&C Red 34 or Orange No. 4, two representative stain solutions. They concluded that the use of H_2_O_2_ plus CoTPP/rGO under photoactivation increased the whitening effect of H_2_O_2_ and decreased the treatment time (Figure 9b). This result might be explained because the active radicals produced by H_2_O_2_ have a short life and so their main effect is to penetrate the tooth structure and then start a radical generation mechanism. When photoirradiation is used, radical generation can be started deeper in the structure. Moreover, photoactivating H_2_O_2_ plus CoTPP/rGO increase the reactions between H_2_O_2_ and stain molecules.

## 4. Limitations on the Use of Graphene

While graphene is one of the most promising materials in nanotechnology, the synthesis on large scale represents an important aspect to be evaluated. Most of the studies described so far have been carried out using small amount of uncontaminated and controlled defect-free samples [153], but in the perspective of using graphene for applications on large scale, defects generated during the production process need to be investigated. Various types of defects can generate spontaneously and their formation is difficult to predict; they depend greatly on the method used for the production [154]. Defects change electronic structure, susceptibility and reactivity of graphene and its derivatives [155]. Most of the production techniques lead to the formation of graphene mixture, that differ in size, shape and number of layer and are often contaminated with hydrocarbons or organic molecules which alter the integration and the compatibility with cells and tissues [153]. These evidences represent a substantial limitation on the study and use of graphene and graphene-based nanomaterials.

Another limitation on graphene biomedical application is the scarce existing information on in vivo toxicity mechanisms and more studies are needed to support safe biological applications of these materials. However, some reports showed that graphene-based nanomaterials mainly accumulate in liver, spleen and lung after intravenous administration [43]. Few studies focused on graphene-based nanomaterials elimination half-life indicating that small-sized nanomaterials present fast elimination [45,156], but further work is needed. In addition, because of its recent discovery, little is known about long-term toxicity of graphene and its derivatives and this issue represents a restriction for clinical approaches.

Therefore, the scarce synthetic control, the high variability in production and the multiple parameters that modulate activity and toxicity of graphene and graphene-based nanomaterials, together with the scarce available studies, are big limitations for large scale application of graphene and its derivatives.

## 5. Conclusions and Future Perspectives

The research on biomedical applications of graphene-based nanomaterials has seen dramatic progress in the last few years. In this review, we focused on the recent advances in dental tissue engineering using graphene and its related nanomaterials. Graphene exhibits numerous outstanding properties. In addition to its exceptional mechanical strength, electrical conductivity and thermal stability, what makes graphene extremely interesting is the possibility to functionalize and combine it with different biomaterials and biomolecules. This confers new properties to existing materials and allows the generation of nanocomposites with enhanced characteristics.

In this review, we discussed recent studies concerning the biocompatibility, cytotoxicity and antibacterial activity of graphene-based nanomaterials both in vitro and in vivo. Then, we analyzed the effect of graphene-based nanomaterials on the adhesion, proliferation and differentiation of stem cells, focusing in particular on stem cells of dental origin. We examined the potential of graphene to promote osteogenic differentiation of stem cells, which is a key point for its future application in dental field. In this context, we presented recent studies employing graphene and its related nanomaterials for surface modification of dental implants or other scaffolds used in dentistry, such as membranes, resins and adhesives. As clearly highlighted in this review, graphene-based nanomaterials have emerged as promising scaffolds for a wide range of biomedical applications and all the presented works seem to agree with the fact that existing dental materials show improved characteristics following the addition of graphene; nevertheless, their behavior is closely dependent on graphene’s physicochemical properties, such as surface functionalization, coating and size.

The increased interest in graphene and its derivatives have led to concerns about the risk of exposure not only to humans but also to the environment. Therefore, evaluation of the safety and potential risks of these nanomaterials is mandatory to ensure the safe use of graphene materials in biomedical applications. However, there is still much investigation to perform in order to assess the potential long-term toxicity of graphene and its composites and their effects on different cells, tissues and organs, including those of oral cavity. Moreover, in-depth studies are necessary to understand cell-signaling, metabolic pathways and osteogenic effects triggered by graphene-based nanomaterials. Overall, we believe that the use of graphene-based nanomaterials in the dental field deserves to be deeply explored as it can lead to even more reliable dental treatments in the near future.

## Figures and Tables

**Table 1:** Examples of different types of membrane used for Guided Bone Regeneration (GBR).

Type	Name	Materials	Characteristics
Non-resorbable	Gore-Tex	Expanded polytetrafluoroethylene	Good space maintainer, handling
	Gore-Tex-Ti	Titanium-reinforced polytetrafluoroethylene	Ideal for ridge augmentation and grafting in bone defects
Resorbable natural	Tutodent	Collagen Type I from bovine pericardium	Resorbable rate: 8–16 weeks
	Osseoguard Flex	Collagen Type I and III from bovine dermis	Resorbable rate: 6–9 months
Resorbable synthetic	Epi-Guide	Poly-d,l-lactic acid	Resorbable rate: 6–12 months
	ResolutAdapt	Poly-d,l-lactide/Co-glycolide	Resorbable rate: 5–6 months, good space maintainer
